# Supplementary material for: Evaluating tuberculosis treatment outcomes and predictors in five Southern African countries: A multi-country cohort analysis
Source: medRxiv. 2026 Mar 20:2026.03.18.26348675. Preprint. [Version 1] doi: 10.64898/2026.03.18.26348675 (PMC13015680; doi:10.64898/2026.03.18.26348675)
Supplement: 1 [file NIHPP2026.03.18.26348675V1-supplement-1.pdf]

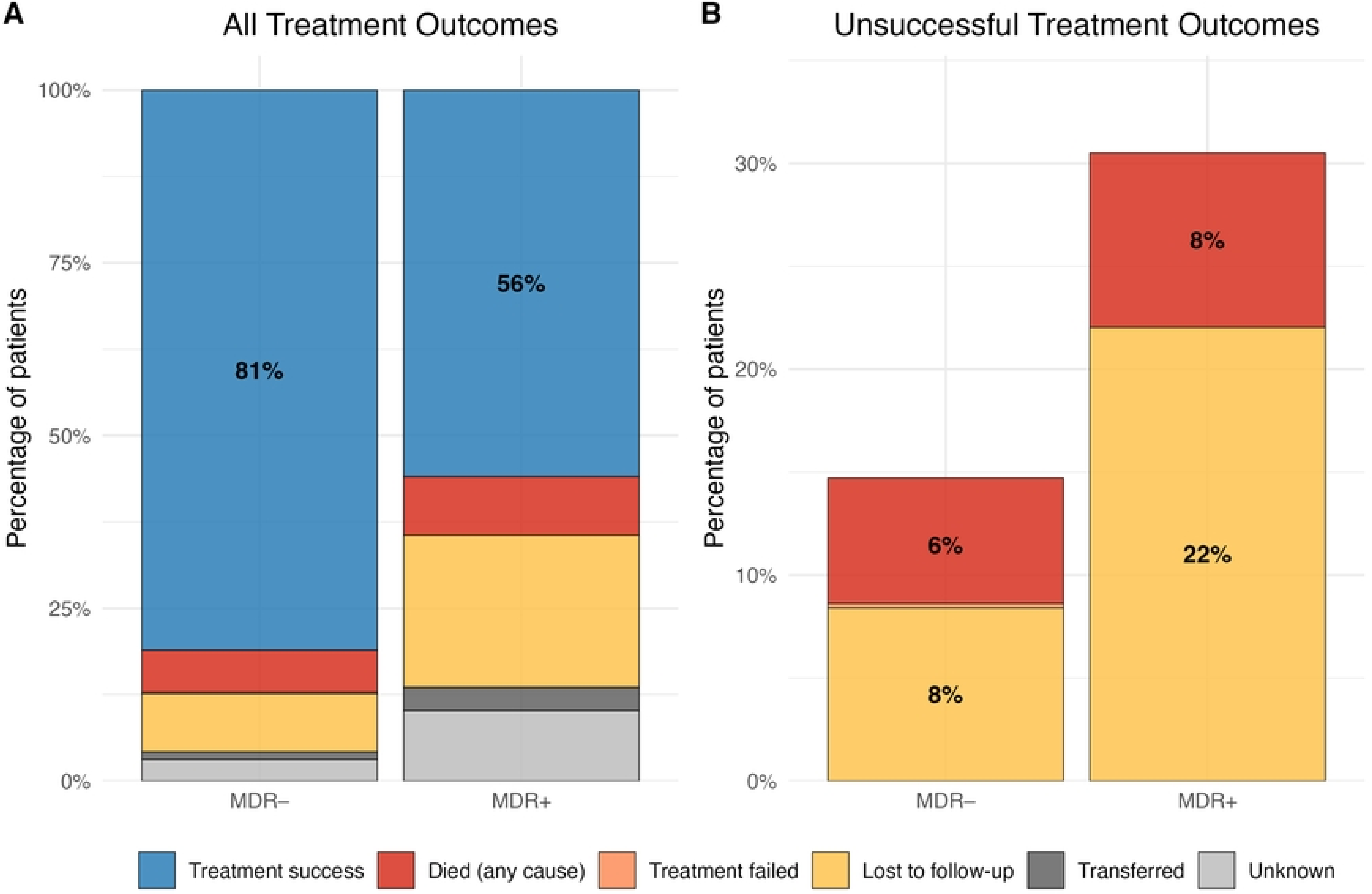

Supplementary Figure 1

medRxiv preprint doi: https://doi.org/10.64898/2026.03.18.26348675; this version posted March 20, 2026. The copyright holder for this preprint (which was not certified by peer review) is the author/funder, who has granted medRxiv a license to display the preprint in perpetuity.
It is made available under a CC-BY 4.0 International license .

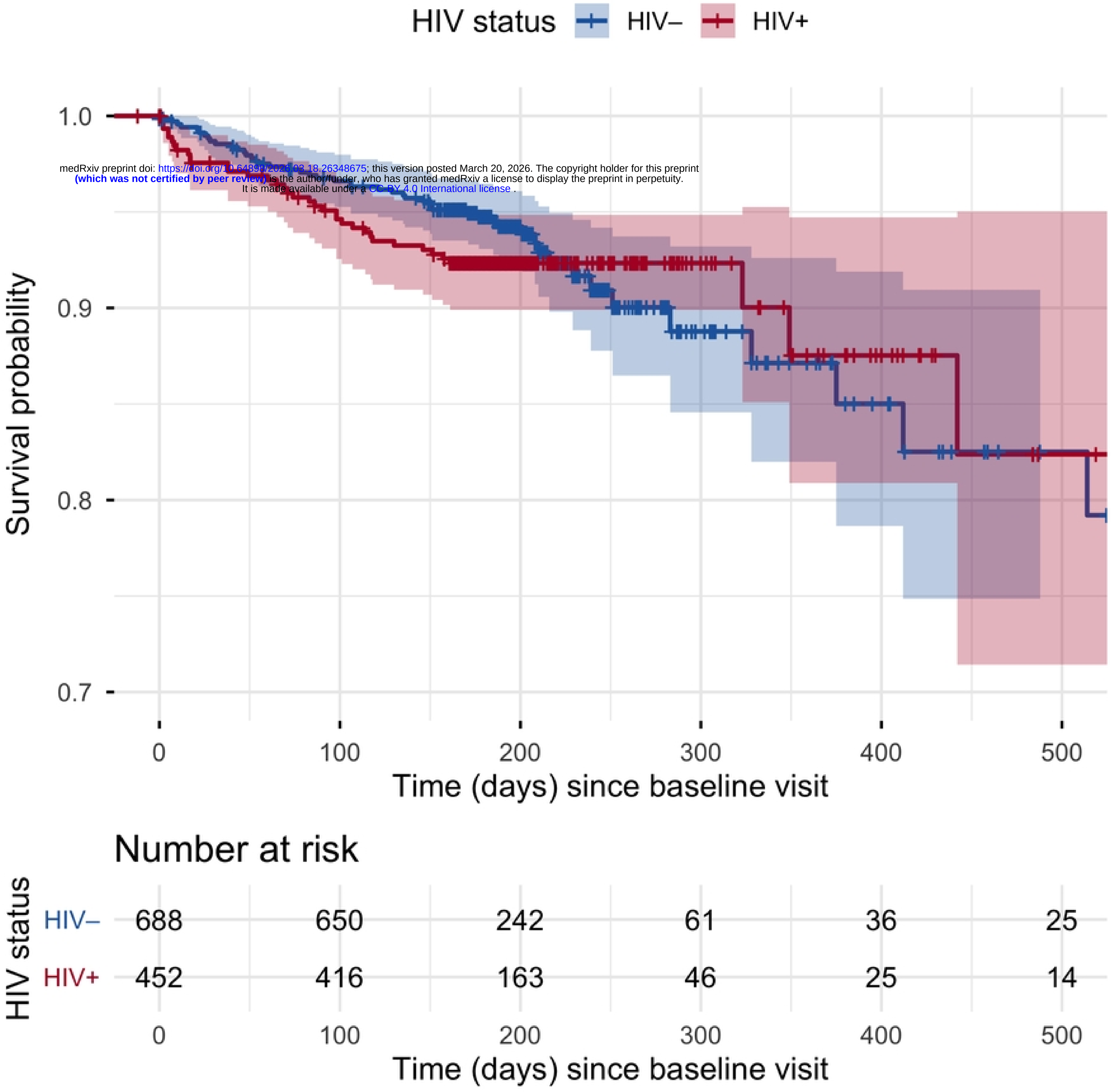

Supplementary Figure 2

medRxiv preprint doi: https://doi.org/10.64898/2026.03.18.26348675; this version posted March 20, 2026. The copyright holder for this preprint (which was not certified by peer review) is the author/funder, who has granted medRxiv a license to display the preprint in perpetuity.
It is made available under a CC-BY 4.0 International license .

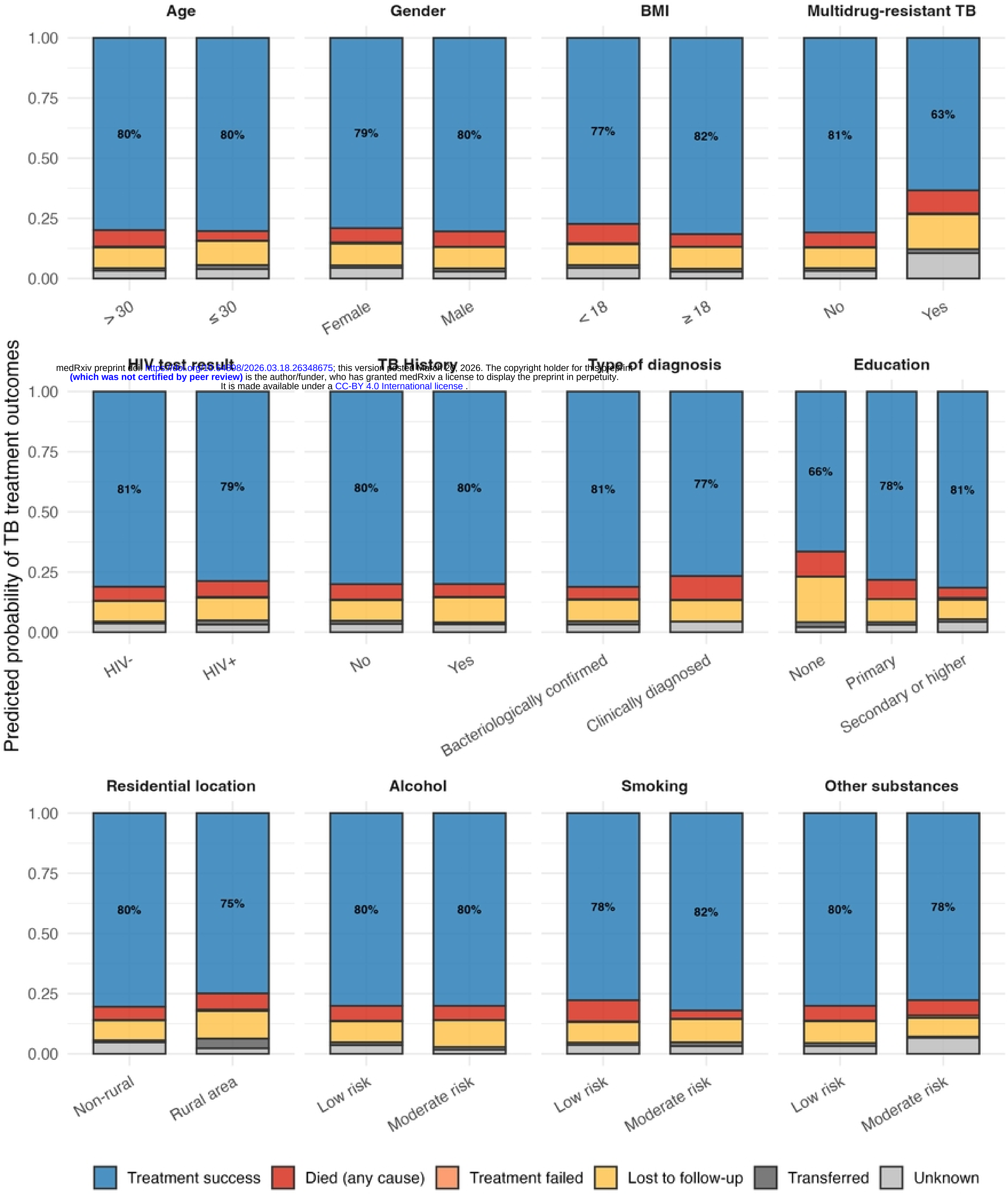

Supplementary Figure 3

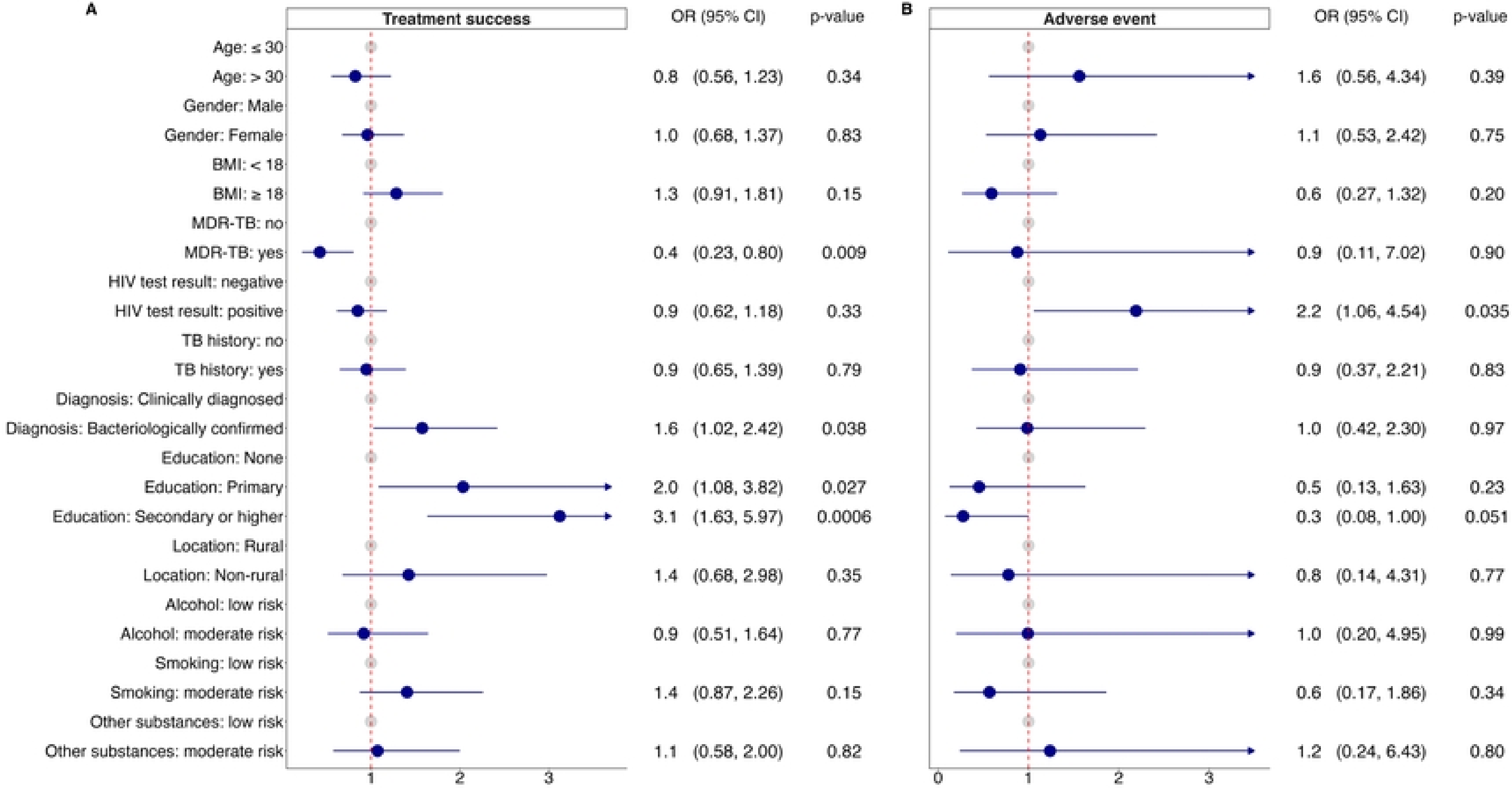

Supplementary Figure 4
